# The Berks County (Pa.) Medical Society

**Published:** 1871-03

**Authors:** 


### THE BERKS COUNTY (PA.,) MEDICAL SOCIETY.

The true friends of the profession—those who adhere to its law and seek to maintain its dignity and honor—are everywhere assailed, within and without, by foes to its progress and its principles. Outsiders—irregulars, or expelled members from our organizations, while illustrating the fact of moral and professional degeneracy, stand as aliens to the rights and principles of ethical law. With such we, or the profession have nothing to do. They, by their action, have chosen the evil rather than the good, and while all sincere members of the profession must lament such action, they can have no interest in them, save only as their deflections from professional rectitude may rebound to the injury of science and the best interests of humanity. But foes within our ranks—those claiming recognition and affiliation with the loyal, while under the guise of conformity to law, not only violate it, but abet those who have been pronounced rebels—should be made to feel that principle still exists and law is yet supreme.

We have indulged in the above reflections in connection with the recent remarkable action of the Berks County (Pa.,) Medical Society. The action of that Society gives token of much professional and moral vitality in their midst. This Society, it seems, had, as a member, a certain professed criminal abortionist. It was found impracticable to reach his criminal transgressions by legal process, and a majority of the Society, fearing they could not secure a two-thirds vote to expel, found the most feasible plan to be to disband. We learn these facts with sincere regret. We regret that any man claiming the exalted position of physician, should so degrade his profession as to engage in a practice derogatory alike to professional and Christian character. We have been taught to hold sacred the life of even the fetus in utero: that no effort should be spared to save it, and no influence weigh for its destruction, save imminent peril on the part of the mother. We regret, also, that bad men should make it necessary for the good to assume the appearance of the aggressive. It is always painful for the good man to collide with the errors and tergivisations of evil doers, and nothing short of duty, and a moral heroism which does not quail before vicious fury, can nerve the arm of virtue, or wield the sword of truth for its vindication.

The dismemberment of this Society seems to have been the only possible escape from the difficulties surrounding it. The abortionist had his friends, professional and otherwise. Here, as everywhere else, the trail of the serpent was visible. There was a local pressure restraining the good, and this pressure was brought to bear upon the Society to such an extent that it was feared many of its members would refuse to lop off the excresence from their midst. The abortionist had his satellites, ready to defile their professional garments at the shrine of mammon, for local interests—unmindful of the principles upon which true science is founded, and willing to stab the profession by bringing reproach upon its integrity and honor. Of all creatures, we most commiserate the human parasite—aimless and senseless, clinging to the arm of vice—lifted into disreputable notice alone by unholy association. That men, who set at defiance all laws, human and divine, could find advocates and defenders, is marvelous; and can alone be understood upon the assumption that humanity is totally, unmistakably depraved.

As a part of the medical history of the day, we give below the preamble and resolutions of the society referred to:

"Dr. Joseph Coblentz moved that the regular order of business be suspended, in order that he might introduce a series of preambles and resolutions; which being granted, he requested the Secretary to read the following:—

"'*Whereas*, The objects of this Society, as defined by its Constitution, are to cultivate the science of medicine and all its collateral branches; to elevate and sustain medical character; to encourage a system of medical etiquette, and to promote mutual improvement, social intercourse and good feeling among the members of the medical profession; and

"'*Whereas*, At its stated meeting, held in this city on the 16th day of August last, this Society, in response to an effort made to preserve its integrity and to maintain the honor and dignity of the profession, did so demean itself, as not only utterly to destroy its usefulness, but also to bring odium and contempt upon the organization itself, as well as disgrace upon the profession in general; and

"'*Whereas*, As a legitimate consequence of such action, in direct violation of Sect 9th, Article 5th, of the Constitution of the Medical Society of the State of Pennsylvania, we must necessarily be deprived of all connection with that Society, and our delegates be excluded from its annual sessions; therefore

"'*Resolved*, That whilst we deplore the necessity that is laid upon us, self-respect, as well as a due regard for the opinion of our professional brethren demands, that we should disband the same, as the only practicable method by which we can be relieved from the unfortunate dilemma in which we have been placed by the disreputable conduct of a portion of our fellow-members, give conclusive evidence of our loyalty to the profession, and be restored to fellowship in the State Society and the American Medical Association.'"

Thus it will be seen that the only means which the good men of the above Society could adopt, of ridding themselves of a disgrace and reproach, was to disband! Why disband? 1st. "To preserve its integrity, and to maintain the honor and dignity of the profession;" "that odium and contempt" should not be brought upon "the organization itself" "as well as disgrace upon the profession in general."

2d. That the Society might not "necessarily be deprived of all connection" with "the Medical Society of the State, and its delegates be excluded from its annual session."

3d. To "give conclusive evidence of their loyalty to the profession, and to be restored to fellowship in the State Society and the American Medical Association."

4th. As the only alternative left them, to cut off the moral and professional putresence from their membership.

To say "well done, good and faithful servants," is but the involuntary plaudit for so noble and righteous an action.

What will the abortionist do? Will he gather his partisans and reorganize the Berks County Medical Society? Will he claim the right to adopt the code of ethics of the American Medical Association, and sound aloud his professional rectitude upon the land and sea—while *sub rosa*, he will still pursue his nefarious calling? Will he claim to be a "bright particular star" in the professional firmament, whose radiance, if lost, will bring "blight and mildew" to science? Will he and his followers demand admittance to associational rights and privileges—and claim recognition and affiliation at their hands? We shall see. Satan, it is said, clothes himself in garments of purity and light—yea, it is even affirmed that he came "among the sons of God to present himself before the Lord." Satan is not yet dead—nor his followers and imitators.

